# Traumatic brain injury reduction in athletes by neck strengthening (TRAIN)

**DOI:** 10.1016/j.conctc.2018.06.007

**Published:** 2018-06-21

**Authors:** Joseph Toninato, Hannah Casey, Mohit Uppal, Tessneem Abdallah, Thomas Bergman, JamesT. Eckner, Uzma Samadani

**Affiliations:** a Hennepin County Medical Center, 701 Park Avenue South, Minneapolis, MN 55415, USA; b University of Minnesota, 100 Church St. SE, Minneapolis MN 55455, USA; c University of Michigan, 1301 Catherine St., Ann Arbor, MI 48109, USA

**Keywords:** Concussion, Football, Sports, Student, Athlete, Brain injury, Head injuries, Neck strength, Prevention, SRC, Sports-related concussion, SCAT5, Sports Concussion Assessment Tool, BAT-L, Boston Assessment of Traumatic Brain Injury Lifetime, TBI, Traumatic Brain Injury, ICC, Intraclasss coefficient, CISG, Concussion in Sports Group, SAC, Standardized Assessment of Concussion, ANOVA, Analysis of variance, MMRF, Minneapolis Medical Research Foundation, IRB, Institutional Review Board

## Abstract

Reporting of sports-related concussions (SRCs) has risen dramatically over the last decade, increasing awareness of the need for treatment and prevention of SRCs. To date most prevention studies have focused on equipment and rule changes to sports in order to reduce the risk of injury. However, increased neck strength has been shown to be a predictor of concussion rate. In the TRAIN study, student-athletes will follow a simple neck strengthening program over the course of three years in order to better understand the relationship between neck strength and SRCs. Neck strength of all subjects will be measured at baseline and biannually over the course of the study using a novel protocol. Concussion severity and duration in any subject who incurs an SRC will be evaluated using the Sports Concussion Assessment Tool 5th edition, a questionnaire based tool utilizing several tests that are commonly affected by concussion, and an automated eye tracking algorithm. Neck strength, and improvement of neck strength, will be compared between concussed and non-concussed athletes to determine if neck strength can indeed reduce risk of concussion. Neck strength will also be analyzed taking into account concussion severity and duration to find if a strengthening program can provide a protective factor to athletes. The study population will consist of student-athletes, ages 12–23, from local high schools and colleges. These athletes are involved in a range of both contact and non-contact sports.

## 1 Background/aims

Recently there has been increased attention to concussion susceptibility in youth contact sports. Sport-related concussions (SRCs) result in approximately 200,000 emergency department room visits every year, 65% of which are pediatric patients ages 5 to 18 [1]. SRCs were determined to be a major health concern as diagnosed concussions have increased by 43% over the past 5 years [2]. More significantly, there was a 71% increase in concussion diagnosis for patients who were 10–19 years old [2]. This rate is even higher among younger populations involved in contact sports. This drastic increase in SRCs has raised concerns for both parents and youth involved in youth contact sports.

While most concussions are mild with patients making full recoveries, sport-related concussions have been associated with drastic short and long-term neuropsychological, neurocognitive, and neurophysiological deficits in youth [7]. Studies have shown that youth that endure one or more concussions are at a substantially higher risk for mental disorders, such as depression, than those who did not experience concussion [6,8]. Furthermore, there is a significant increased risk of suicide and suicidal tendencies in people who were exposed to concussion in adolescence [6]. Not all side effects and prognoses of incurring multiple concussions over a person's lifetime are known due to most studies having ascertainment biases, small study groups, and no controls. However, it is clear that efforts to reduce cumulative impacts would likely be beneficial.

Published literature reveals the role that neck strength plays in contact sports and concussion diagnosis. Hildenbrand et al. conducted a study in 2013 to investigate the average neck strength of high school athletes in order to help determine how neck strength could relate to the biomechanics of certain types of muscle or neurological injuries [3]. This study used a multi-cervical device that measured isometric neck strength. Using a cohort size of 149 high school and college athletes (77 male and 72 female), the investigators determined that, on average, males had stronger necks than females who played contact sports at both the high school and collegiate levels [3].

In addition, Eckner et al. looked at how neck-strength affected the kinematic response of the head to impulsive loads in a controlled lab environment [4]. The investigators measured isometric neck strength in 46 subjects, and measured the head's kinematic response to impulsive loads, simulating impacts to the head. This study concluded that greater neck strength was associated with a decreased kinematic response of the head to controlled impulsive loading [4]. Collins et al. developed a novel neck strength measurement tool and conducted a study to assess its feasibility for use by athletic trainers in student athletes and its potential as a concussion risk prediction tool. In the course of this study, the research team found that neck strength to be a predictor of concussion risk, and that neck strengthening may provide a protective factor to student athletes. Their results demonstrated a statistically significant correlation between weaker neck strength and increasing susceptibility to concussion in high school contact sports. It was determined that every 1 pound increase in neck strength contributed to a 5% decrease in odds for a concussion event occurring [5].

Taking all of this into consideration, the purpose of TRAIN is threefold.

Aim 1 Understand the relationship between neck strength and risk of SRC in student athletes.

Hypothesis 1 Greater neck strength will correlate with lower risk of SRC

Aim 2 Determine the effectiveness of a simple neck strengthening program to reduce the risk of SRC.

Hypothesis 2 Greater improvement in neck strength will correlate with greater reduction of SRC risk

Aim 3 Test the feasibility of implementing such a program in a large population of student athletes.

Hypothesis 3 With proper monitoring and school participation, neck strengthening can be implemented on a large scale to student athletes

## 2 Study methods

### 2.1 Protocol overview

The study population will consist of student-athletes from six Minnesota high schools and colleges. Subjects will partake in a variety of both contact and non-contact sports. Participating subjects will attend a baseline event where their neck strength and girth will be measured. These individuals will also complete the Sports Concussion Assessment Tool (SCAT5), eye tracking, and the pre-military portion of the Boston Assessment of Traumatic Brain Injury-Lifetime (BAT-L). Following baseline assessments, student-athletes will follow a neck-strengthening exercise regimen. Neck strength will be re-evaluated at 6-month intervals while SCAT5 and eye tracking are done yearly and in the event of a concussion.

### 2.2 Baseline assessment

Following a consent/assent meeting with the athletes and their parents, subjects will attend a baseline event which included various assessments. First, subjects will complete a brief demographic survey about information regarding birth date, year in school, sport played, and the number of concussions the athlete has had in his or her lifetime. If the participant answers with a number greater than 0 to this last question, they will then complete the pre-military portion of BAT-L, filling out more information regarding their three worst traumatic brain injuries (TBIs). BAT-L asks for their age during each incident, mechanism of injury and symptoms experienced after the TBI.

Next, subjects will have their neck strength measured using a MicroFet2 dynamometer mounted onto either a squat rack or a pull-up bar using the method described in the outcome measurement section below. The strength of their flexion, extension, and right and left lateral flexion will be measured. Then, their head and neck girth will be measured in centimeters. Head girth will be defined as the largest part of their head right above their eyebrows and ears. Neck girth will be measured directly below their laryngeal prominence (commonly known as an ‘Adam's apple’). Finally, the subjects will be eye-tracked using an automated algorithm and asked to complete the SCAT5 assessment. Both of these assessments will be used as concussion outcome measures.

### 2.3 Re-evaluation/concussion protocol

As stated above, neck strength measurements will be re-evaluated every six months following the baseline event. The SCAT5 and eye tracking will be re-evaluated every year following the baseline event. If at any point during the study a participant experiences a concussion, the SCAT5 will be immediately conducted by athletic trainers on-site, or within 24 h by research staff, and research personnel are contacted by the school's athletic director. Research personnel will then meet with the student-athlete to complete the SCAT5 and eye tracking weekly until symptoms are resolved and the athlete is cleared to return to normal activity by his or her physician. The SCAT5 and eye tracking assessments will be compared to baseline as measures of TBI severity in subjects, as well as track progress towards recovery.

### 2.4 Neck strengthening exercises

Subjects and their teams' coaches will be instructed on a manual-resistance-based neck strengthening exercise program, to be performed twice a week on non-consecutive days. This instruction will include the proper form for each exercises, the proper resistance methods, and the proper frequency and set/rep scheme. The exercise program is a modified version of that previously described by Eckner et al. [23] that will be performed in peer-peer or peer-coach pairs. In addition to initial in-person instruction, demonstration videos will also be provided online at pconsstudy.com. The coaches will lead the exercises as part of the teams' warm up, or regular strength training sessions, during the competitive season. Other arrangements may be made given extenuating circumstances such as a small subject population on the team. During the off-season, subjects will perform the exercises at the same time of day, on the same days of the week as they did during the competitive season. Weekly questionnaires will be sent via emails to assess subject compliance. Included will be questions about frequency of exercises, when they were done, rep and set scheme, whether they were led by a coach, how many other subjects did them at the same time, if they were done as part of a larger strengthening program or part of a warm up, etc.

### 2.5 Outcome measures

#### 2.5.1 Neck strength measurements

**Fig. 1:**
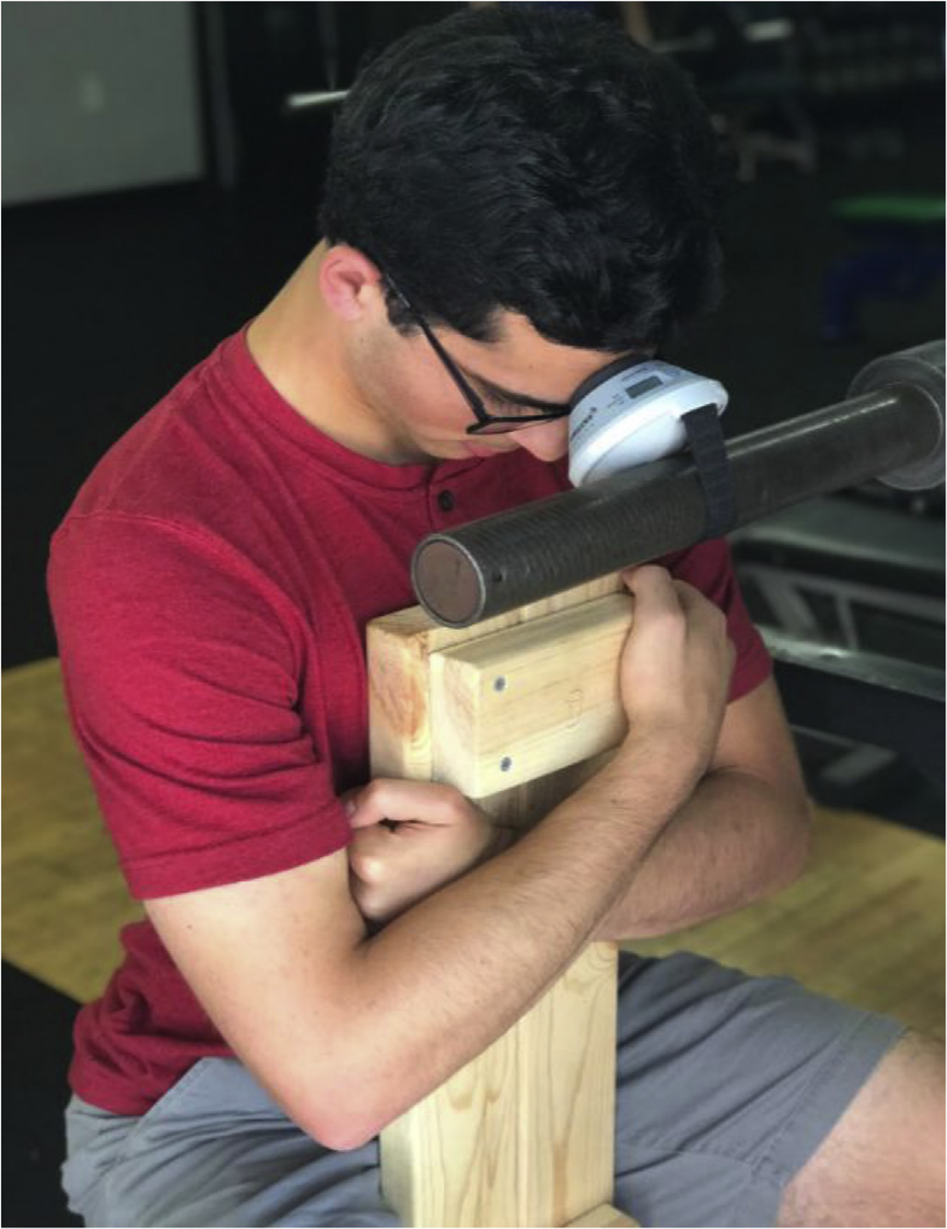
Image of flexion strength being measured using the MicroFet2 dynamometer and custom built seat.

A novel method of neck strength measurement was created for use in this trial, using the MicroFet2 handheld dynamometer to determine the peak force in pounds of a subject's neck muscles in each of the stated directions. A custom chair with multiple vertical inserts was built for subjects to sit on to accommodate varying height, the visible wooden portion seen in Fig. 1. These inserts were created in order to isolate the subject's neck as the only mover applying force. The MicroFet2 is placed on retractable pull up bar, or a barbell in a squat rack, and positioned in front of the chair. The height of the dynamometer is then adjusted so it sits just in front and below the contact point of the subject's head: the forehead, the occipital prominence, and positioned in front of the chair. The height of the dynamometer is then adjusted so it sits just in front and below the contact point of the subject's head: the forehead, the occipital prominence, and between the ear and temple for flexion, extension, and lateral flexion respectively. Neck positioned in front of the chair. The height of the dynamometer is then adjusted so it sits just in front and below the contact point of the subject's head: the forehead, the occipital prominence, and between the ear and temple for flexion, extension, and lateral flexion respectively. Neck strength is measured by the subject sitting on the chair facing toward the dynamometer, away from it, or to the left or right depending on which direction is being measured. The assessor then places the correct vertical insert into the slot, so the insert lines up with the subject's mid-chest. For forward and lateral flexion, the subject tightly hugs the insert to ensure their body is tight to it. The subject then flexes or extends their head into the MicroFet2 as hard as they are able for 3 s. This is repeated for a total of three times per metric, and averages are calculated for each. In this way, peak forward flexion, lateral flexion, and extension force of the neck are measured.

To ensure accuracy and reliability of this novel protocol, two small trials were performed prior to subject recruitment. The first of these verified the ability of each MicroFet2 to measure force accurately and consistently with one another. This consisted of three objects of differing weights, and all three dynamometers Table 1 shows the results of this trial. The second trial involved four testers measuring the forward flexion, the most variable direction, of eight individuals over four days in order to test inter-rater reliability. A single measurement, absolute-aggrement, 2-way mixed-effects model intraclasss coefficient (ICC) was calculated and found to be acceptable at 0.78, with the ICC between the first two days being 0.84 and the ICC between the last two days being 0.96. Table 2 summarizes these results.

**Table 1:** Table represents MicroFet2 dynamometer measurements of fixed weights, testing for measurement accuracy. The inter-device correlation was ∼1.

Item Weight (lbs)	Device 1 (lbs)	Device 2 (lbs)	Device 3 (lbs)
3.95	3.9	3.9	3.9
8.71	8.7	8.6	8.7
141.2	141.1	141.2	141.1

**Table 2:** Table represents MicroFet2 dynamometer measurements of the same subjects on different days, testing for measurement reliability. The ICC was found to be 0.78.

Subject Number	Day 1 (lbs)	Day 2 (lbs)	Day 3 (lbs)	Day 4 (lbs)	Mean (lbs)	SD (lbs)
1	15.9	16.9	16.3	16	16.28	0.45
2	10.0	12.7	13.6	14.2	12.63	1.86
3	10.9	12.9	–	–	11.9	1.41
4	9.6	–	14.9	15.8	13.43	3.35
5	21.2	23.3	29.5	29.7	25.93	4.33
6	20.1	26.3	20.5	22.6	22.38	2.84
7	6.6	–	7.8	8.0	7.47	0.76
8	11.6	15.9	18.3	22.7	17.13	4.64

#### 2.5.2 Sport concussion assessment tool 5th edition (SCAT5)

The Sport Concussion Assessment Tool 5th Edition (SCAT5) will be used to determine concussion severity and duration. The original SCAT was first designed in 2004 during a meeting of the Concussion in Sports Group (CISG) [9]. The tool was created in order to help quickly evaluate a person for an SRC. Since its creation, the CISG has created 4 versions of this tool- SCAT, SCAT2, SCAT3, and the current SCAT5. Each iteration has been widely used as “sideline” assessments [10,12], and often recommended for use due to its wide range of assessments that are continuously under review to ensure relevance to SRCs [9,11].

The current edition, the SCAT5 includes two well-known concussion assessments: the Standardized Assessment of Concussion (SAC) and a symptom checklist similar to the Post Concussion Scale. The SAC comprises of an orientation assessment, both immediate and delayed word recall tests, as well as a concentration screen. Studies have shown that the overall SAC score, immediate memory score, and concentration score of concussed athletes is significantly lower than their own baseline SAC score, and the concussed athletes' overall SAC score and immediate memory score is lower than those of their non-concussed teammates [13,14].

Symptom and symptom severity assessments have also been used as a way to gauge severity of concussion, as well as aid in post-concussion treatment. Babl et al. found that the symptom questionnaire in the SCAT3 was able to differentiate between concussed and non-concussed children and adolescents due to the higher number and severity of symptoms reported in concussed individuals [15]. Similar findings have been reported for athletes within the same range [16]. In addition, symptom severity has been found to be associated with clinical outcome [17]. A broad review of concussion recovery articles by Iverson et al. found that many studies reported worse outcomes, longer recovery times and more severe trauma, were associated with more severe reported symptoms post-injury [17].

Due to the breadth and validity of the above assessments and its use of a balance exam, the SCAT5 is used in this study for the detection of sports-related concussion in our study population.

#### 2.5.3 Boston Assessment of Traumatic Brain Injury- lifetime (BAT-L)

History of TBI will be evaluated using the Boston Assessment of Traumatic Brain Injury-Lifetime (BAT-L). This is a semi-structured clinical interview used to classify TBIs in veterans through questions regarding pre-military, military, and post-military events throughout an individual's lifetime. Given the prevalence of sports-related concussions, the pre-military portion of BAT-L is used to classify previous TBIs in our subjects. The military and post-military portions of this survey were deemed irrelevant to young athletes who are not participating in combat. In a study validating BAT-L, it was found to strongly correspond with the Ohio State University TBI Identification method, an assessment frequently used to classify TBIs in athletes [17]. In comparison, the pre-military portion of the BAT-L is more detailed.

Zero is given to subjects without any TBIs. A score of 1 through 3 is assigned to subjects with mild TBIs, with further categorization of grade I, grade II, and grade III. A score of 4 is given to a moderate TBI and a score of 5 is given to a severe TBI. The BAT-L was also validated against the VA TBI assessment and found to have moderate correspondence with 0.85 sensitivity and 0.82 specificity [18].

#### 2.5.4 Eye tracking

Eye tracking will help evaluate concussion severity and duration. For the study, this will be done using the SR Research Eyelink 1000, which consists of a screen, an infrared eye tracker, and a mount to stabilize the subject's head. Once the subject is seated comfortably and properly with their head in the mount, a 220-s video will play on the screen. This video only takes up a fraction of the screen and moves around its square perimeter. The infrared camera will track the movement of the subject's eyes independently. This data is then analyzed using novel algorithms to yield 89 metrics involved in eye movement and tracking. This method has been shown to detect disconjugate eye movement associated with head trauma [21], as well as changed ratios of vertical to horizontal eye movements in patients with cranial nerve palsies [22].

**Fig. 2:**
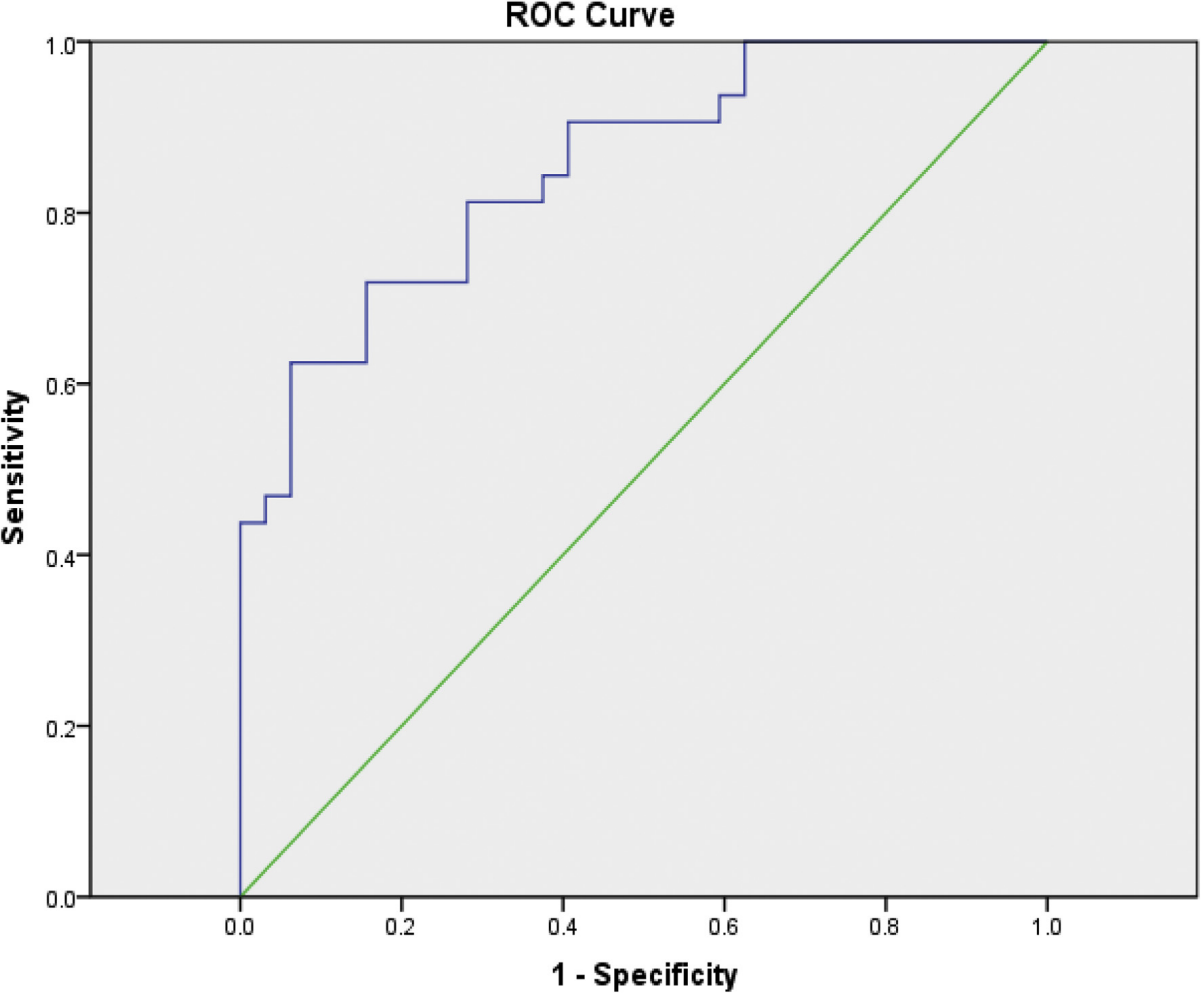
Receiver operating curve for eye tracking as a method of diagnosing concussion.

Eye movement dysfunction is frequently associated with concussion [19], and is posited to serve as a screening diagnostic [20]. In unpublished preliminary trials, eye tracking was able to detect concussion with reasonable sensitivity and specificity (Fig. 2). As such, using this protocol at baseline and for concussion assessment will not only allow us to better understand severity of head trauma, but also give us an opportunity to validate eye tracking as a simple sideline test for concussion.

#### 2.5.5 Data analysis

Repeated-measures analysis of variance (ANOVA) will be run comparing neck strength between concussed and non-concussed subjects within various sub populations defined by age, sex, sports, etc., in order to analyze the first two aims. Alongside this, linear and logistic regression models will be implemented as secondary analyses to establish relationships between neck strength and other functional outcomes tested during the course of the study such as eye tracking, SCAT5 scores, and history of TBI. To meet the third aim, we will monitor compliancy to the neck strengthening by measuring average change, and seeing if it is significant. Coaches and athletic staff will also give input on how to improve efficiency of implementation.

## 3 Limitations

Every measuring method has a degree of error, particularly when done by a human. Differences resulting in how study staff set up our neck strength measurement equipment, how a subject moves and follows instructions, and other factors do present a limitation to this study. In our preliminary trial, we found the error measured by ICC to be acceptable however. The degree of difficulty of the exercises will also most likely vary due to different people leading the exercises. The coaches for each team, as well as the subjects, will be instructed on proper form and technique, but small variations from length of exercise, to how much resistance is applied will inevitably differ. The largest limitation to our study will be compliance to the neck strength exercises. Due to the size of the study population compared to the size of our staff, team and school buy in will be paramount. In addition to the measures described in section 2.4, we are looking into using the student captains as drivers for each team to be compliant.

## 4 Ethics for human subjects

As this study involves human subject, approval of this study was given by the Institutional Review Board of the Minneapolis Medical Research Foundation before any study procedures took place. In addition, all possible subjects are met with by study staff prior to all assessments in order to obtain informed consent. This involves outlining the study (rationale, protocols, outcome measurements, etc.), as well as providing each possible subject with information about the researchers, research institutions, all risks and benefits of the study, confidentiality of any and all data collected, costs and compensations, conflicts of interest associated with the primary investigators, and the freedom to participate and withdraw at any time. Each subject receives a copy of these consent documents, and the original is stored for lab records. All of these actions are in line with MMRF, state, and federal guidelines for use of human subjects.

## Funding

Funding for this study comes from the Minnesota Spinal Cord Injury and Traumatic Brain Injury Research Grant, the Rockswold Kaplan Endowment Fund, and Charlene's 5 k Dog Run. Funds from each has contributed to all aspects of study expenses including salaries, travel, and equipment.
